# Acceptability of transdermal antipsychotic patches by patients who refuse oral medication and their effectiveness in preventing recurrence of delirium: a retrospective observational study

**DOI:** 10.1097/YIC.0000000000000428

**Published:** 2022-08-12

**Authors:** Kotaro Hatta, Chie Usui, Hiroyuki Nakamura

**Affiliations:** a Department of Psychiatry, Juntendo University Nerima Hospital, Tokyo; b Department of Environmental and Preventive Medicine, Kanazawa University Graduate School of Medical Science, Kanazawa, Japan

**Keywords:** antipsychotic, blonanserin, delirium, patch, prevention, refusal, transdermal

## Abstract

Injectable antipsychotics had been used for patients who refuse oral medications in delirium practice. The objectives were to investigate acceptability of transdermal antipsychotic patches by patients who refuse oral medications and their effectiveness in preventing recurrence of delirium. In this retrospective observational study, data were collected between October 2019 and December 2021. The sample was represented by patients hospitalized because of acute diseases or elective surgery who had delirium on the night before the consultation and had refused oral therapy after consultation. Delirium has been diagnosed according to the Diagnostic and Statistical Manual of Mental Disorders, Fifth Edition. Instead, a transdermal patch of blonanserin, a second-generation antipsychotic drug, was tried. The primary outcome was the rate of patients who accepted it. The secondary outcome was recurrence rates of delirium. As much as 95.1% of patients who refused oral medications (98/103 patients) accepted to receive the transdermal patch. Of these, 24 patients developed delirium again, whereas all five patients who refused it developed delirium again [24.5% (24/98) vs. 100% (5/5); *P* = 0.0014]. The present findings suggest that transdermal antipsychotic patches are more likely to be accepted by patients who refuse oral medications. Prospective studies are needed.

## Introduction

Delirium represents an acute change in cognition with altered consciousness and impaired attention that fluctuates over time (American Psychiatric Association, 2013; European Delirium Association and American Delirium Society, 2014). Multiple studies have found older age to be an independent risk factor for delirium among hospitalized, medically ill older adult patients, with an increased delirium risk from 3% for those less than 65 years old to 36% for patients 75 years and older (Pendlebury *et al.*, 2015; Pinho *et al.*, 2016; Maldonado, 2018). With the increase in the aged population, further increases in delirium seem likely. Therefore, attention has been increasingly paid to delirium prevention. There is moderate-certainty evidence regarding the benefit of multicomponent nonpharmacological interventions in hospitalized adults (Burton *et al.*, 2021). Regarding pharmacological interventions, our randomized clinical trials have shown effects of a melatonin receptor agonist and an orexin receptor antagonist on delirium prevention (Hatta *et al.*, 2014; Hatta *et al.*, 2015; Hatta *et al.*, 2017). Recent meta-analyses support these findings (Xu *et al.*, 2020; Khaing and Nair, 2021). Furthermore, our prospective real-world data showed preventive effects of these drugs on the recurrence of delirium (Hatta *et al*., 2020). However, these drugs are not usable for patients who refuse oral medications, as they have only an oral formulation. Refusal of oral medications is unmet need of older people with delirium, especially, with comorbid behavioral and psychological symptoms of dementia. In that case, a frequent measure has been an antipsychotic injection such as intravenous or intramuscular haloperidol, which has some benefits of prophylactic use (Wang *et al.*, 2012). In a recent meta-analysis of the pharmacological prophylactic measures for postoperative delirium, dexmedetomidine, olanzapine, and risperidone have shown higher efficacy than other drugs (Liu *et al.*, 2019). However, dexmedetomidine hydrochloride injection is only indicated for sedation of nonintubated patients before and/or during surgical and other procedures (prescribing information of dexmedetomidine hydrochloride injection). Risperidone is not available in any formulation other than oral and long-acting antipsychotic injection, making it inaccessible to patients who refuse medication. Olanzapine can accommodate patients who refuse medication because of the availability of intramuscular formulations, but their coercive actions often disrupt the patient-provider relationship. Therefore, we have been looking for a noninjection method that does not involve coercion.

The Japanese Ministry of Health, Labour and Welfare approved a transdermal patch of antipsychotic drug, which is blonanserin, in September 2019 for the treatment of schizophrenia in adults (LONASEN Tapes package insert). The transdermal patch of blonanserin has been expected to enhance treatment adherence for long course of schizophrenia treatment. Moreover, we noticed that patients who refused oral medications unexpectedly and frequently accepted to receive the transdermal patch. So, we began to use this patch for not only acute schizophrenia patients but also patients who had the hyperactive subtype of delirium on the night before the consultation and refused to take medicine orally after the consultation.

The objectives were to investigate acceptability of transdermal antipsychotic patches by patients who refuse oral medications and their effectiveness in preventing recurrence of delirium.

## Methods

### Study design

This is a retrospective, observational study, and all study protocols were approved by the Research Ethics Committee, Faculty of Medicine, Juntendo University (reference number: E21-0257). All methods were carried out in accordance with the Declaration of Helsinki and the Ethical Guidelines for Medical and Biological Research Involving Human Subjects. The approved protocol did not require informed consent from the patients, as the design was retrospective, and because the data remained anonymous and were analyzed in aggregate. Instead, we posted a notice in the hospital providing a means for the patients to opt out. We reported the study according to the STROBE statement.

### Setting

This study was conducted by trained psychiatrists as consultation-liaison psychiatric services in a university general hospital located in Tokyo. The period of data collection was 27 months (from 1 October 2019 to 31 December 2021), and that of follow-up was 2 weeks.

### Participants

Patients were hospitalized because of acute disease or elective surgery and had the hyperactive subtype of delirium on the night before the consultation. They were introduced into consultation-liaison psychiatric services by the physician/nurse in charge and were assessed by two trained psychiatrists (K.H. and C.U.). Then, psychiatrists recommended patients to take delirium-preventive sleeping pills such as a melatonin receptor agonist and orexin receptor antagonists (Hatta *et al.*, 2014; Hatta *et al.*, 2015; Hatta *et al.*, 2017) for the following nights. Among them, eligible patients were those who refused such oral medications. Alternatively, psychiatrists recommended a transdermal patch of blonanserin. Patients received a verbal explanation about the patch and could reject the recommendation. All patients received multicomponent, nonpharmacologic delirium prevention interventions by nurses (Ogawa *et al.*, 2019). When patients developed delirium again, an injection of haloperidol was used as needed.

### Variables and measurement

The patients’ demographic and clinical characteristics, including age, sex, BMI, presence or absence of dementia/MCI, previous delirium, admission diagnosis, habitual use of alcohol, habitual use of benzodiazepine receptor agonists, opioids, corticosteroids, scores of Clinical Dementia Rating (Hughes *et al.*, 1982), Charlson Comorbidity Index (Charlson *et al.*, 1987), and Eastern Cooperative Oncology Group performance status (Falkson *et al.*, 1980), the serum level of C-reactive protein at the beginning of treatment, the cause of delirium according to the Delirium Etiology Rating Checklist (Trzepacz *et al.*, 2010), and adverse events, were collected from medical records. Scores of sleep–wake cycle disturbances according to item 1 of the Delirium Rating Scale-Revised-98, from 0 (not present) to 3 (severe disruption), were collected from medical records at the beginning of treatment and after 2 weeks (Trzepacz, 1999; Kato *et al.*, 2010).

The primary outcome was the rate of patients who accepted to receive the transdermal antipsychotic patch among patients who refused oral medications. The secondary outcome was the rate of patients who developed delirium again during the first 2 weeks among them, compared with the rate in patients who refused both oral and transdermal formulations. A transdermal patch of blonanserin 20 mg was given at 15:00 until patients came to accept oral medications. The Diagnostic and Statistical Manual of Mental Disorders, Fifth Edition (DSM-5) criteria were used to diagnose delirium by trained psychiatrists (K.H. and C.U.) at the consultation and every morning round.

### Statistical analyses

As there was no prior study about the effect of transdermal antipsychotic patches on delirium in patients who refused oral medications, we could not set a particular study size. Instead, we tried to collect consecutive cases of more than 100.

To examine whether the transdermal patch of blonanserin would help prevent recurrence of delirium in patients with delirium on the night before consultation, we compared patients who received it with those who refused it. For patients who were discharged before the end of the 14-day observational period, data during hospitalization were utilized.

Data were collected on standardized forms, and statistical analyses were performed using SPSS (version 28-J; IBM Japan, Tokyo, Japan). Differences between categorical variables in the patients’ demographics and clinical characteristics were calculated using Fisher’s exact test. Differences between sequential variables were calculated using unpaired *t*-tests (with Welch’s correction if applicable). If data were not sampled from Gaussian distributions, a nonparametric test (Mann–Whitney test) was used. We planned to construct multivariate logistic regression models to control for risk factors in estimating independent associations between the effects of the transdermal patch of blonanserin and the outcome of delirium as an exploratory analysis. All statistical tests were two-tailed. Differences were considered statistically significant at *P* < 0.05.

## Results

**Fig. 1:**
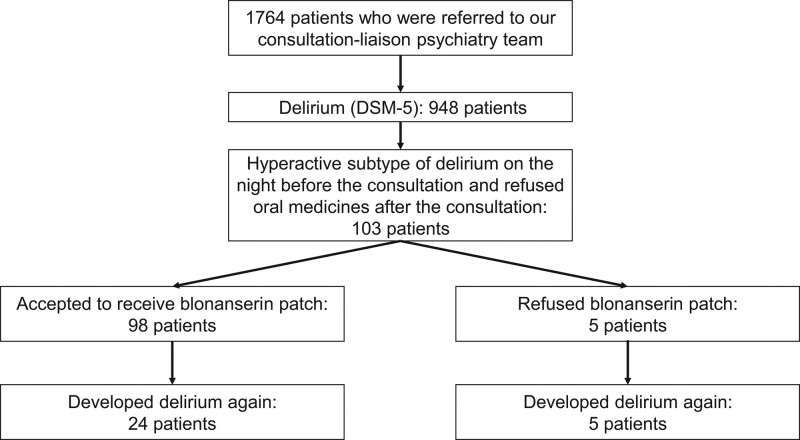
Flow chart.

Among 1764 patients who were referred to our consultation-liaison psychiatry team during the study period, 948 patients were diagnosed with delirium defined by the DSM-5. Of these, 103 patients presented with a hyperactive subtype of delirium on the night before the consultation and refused oral medications to prevent delirium for the following night. The mean age was 78.8 (SD, 10.3), and 64 patients (62.1%) were men. Of these, 98 patients (95.1%) accepted to receive the transdermal patch, whereas five patients refused it (Fig. 1). There were no significant differences in demographic characteristics between the groups (Table 1).

**Table 1:** Baseline characteristics and clinical outcomes among patients who had delirium on the night before the consultation

Variable	Patients who accepted blonanserin patch	Patients who refused blonanserin patch	*P* value
Number of patients	98	5	
Age, mean (SD), years	79.2 (10.3)	72.0 (8.2)	0.13
Male, No. (%)	60 (61.2)	4 (80.0)	0.65
BMI (SD)	21.1 (3.5)	20.7 (3.5)	0.83
Dementia/MCI, No. (%)	81 (82.7)	4 (80.0)	1.00
Clinical dementia rating (SD)	1.36 (1.12)	1.20 (0.84)	0.75
Previous delirium, No. (%)	6 (6.1)	0 (0)	1.00
Admission diagnosis, No. (%)			
Infection (systemic)	29 (30.0)	0 (0)	0.32
Cerebrovascular	14 (14.3)	1 (20.0)	0.55
Organ insufficiency (cardiac)	13 (13.3)	1 (20.0)	0.53
Neoplasm (systemic)	12 (12.2)	2 (40.0)	0.14
Neoplasm (intracranial)	6 (6.1)	0 (0)	1.00
Other	24 (24.5)	1 (20.0)	1.00
Surgery, No. (%)	12 (12.2)	0 (0)	1.00
Habitual use of alcohol, No. (%)	12 (12.2)	2 (40.0)	0.14
Habitual use of benzodiazepine receptor agonists, No. (%)	2 (2.0)	0 (0)	1.00
Prescribed opioid, No. (%)	12 (12.2)	2 (40.0)	0.14
Prescribed corticosteroids, No. (%)	11 (11.2)	0 (0)	1.00
C-reactive protein, mean (SD), mg/dl^a^	6.71 (9.07)	5.42 (7.68)	0.75
Fluctuation of SpO_2_, No. (%)	21 (21.4)	0 (0)	0.58
Charlson Comorbidity Index, mean (SD)	3.31 (2.24)	4.80 (2.59)	0.15
Performance status, mean (SD)	3.60 (0.55)	3.60 (0.89)	1.00
Sleep–wake cycle disturbance at the beginning of intervention, mean score (SD)^b^	1.60 (0.74)	1.60 (0.89)	0.88
Sleep–wake cycle disturbance at the end of intervention, mean score (SD)^b^	0.49 (0.69)	0.80 (0.84)	0.39
Recurrence of delirium, No. (%)	24 (24.5)	5 (100)	0.0014
Adverse event, No. (%)	2 (2.0)	0 (0)	1.00

a Values of C-reactive protein were serum levels.

b Sleep–wake cycle disturbance was evaluated according to the item 1 of Delirium Rating Scale-Revised-98, 0 (not present) to 3 (severe disruption of sleep–wake cycle).

Patients who received the transdermal patch of blonanserin developed delirium again less frequently than those who refused it [*n*/*N* = 24/98 (24.5%) vs. 5/5 (100%), odds ratio, 0.030; 95% confidence interval, 0.0016–0.56; *P* = 0.0014]. We did not construct multivariate logistic regression models because the recurrence rate of delirium in patients who refused the transdermal patch was 100%, and because no significant differences were found in demographic characteristics between the groups. Patients who developed delirium again received haloperidol injections as needed. Adverse events were observed in two patients who received the transdermal patch of blonanserin (1.9%): one was sleepiness, and another was slight extrapyramidal symptoms.

## Discussion

The present study found that a high percentage of patients with delirium on the night before the consultation accepted the transdermal antipsychotic patch despite their refusal of oral medication. The reasons for the acceptance of the transdermal patch by these rejective patients might have been as follows. Patches are visible unlike oral medication. So, patients can remove them when they fill with apprehension against the drugs. To our knowledge, there is no study of transdermal patches regarding refusal of oral medication. In an open-label multicenter study about long-term safety and efficacy of blonanserin transdermal patch, patients’ attitudes to the patch were reportedly positive with respect to continuing treatment, compared with taking tablets (Iwata *et al.*, 2020). Thus, transdermal patches might have advantages of adherence or a patient-friendly character.

Second, the present study has shown a significant difference in the incidence of recurrence of delirium between patients who received the transdermal patch of blonanserin and those who refused it. This suggests that the transdermal patch of blonanserin is effective in preventing recurrence of delirium. Because of the noninvasive character, the transdermal patch of blonanserin may be a treatment choice regarding prevention of recurrent delirium in patients who refuse oral medication prior to an antipsychotic injection.

Transdermal antipsychotics have the potential to enhance treatment adherence, so the US Food and Drug Administration approved transdermal asenapine in 2019 for the treatment of schizophrenia in adults (Musselman *et al.*, 2021). Its possible place in real-world practice has been sought. The present findings showing the transdermal patch of blonanserin in preventing the recurrence of delirium suggest one of possible places for antipsychotic transdermal patches.

A major limitation of this study is retrospective design. In addition, these results might be related to the fact that Japanese people are accustomed to using patch products, such as applying them frequently for pain. The data were from a university general hospital. To generalize findings, studies conducted in other countries and other clinical settings are needed.

In conclusion, the present findings suggest that transdermal antipsychotic patches are more likely to be accepted by patients who refuse oral medications. Furthermore, the transdermal patch of blonanserin might be effective in preventing recurrence of delirium in patients who had delirium on the night before the consultation. As transdermal patches appear to be superior to involuntary injections in maintaining a positive relationship between the patient and the medical staff, they may be an effective tool in practice. Prospective studies are needed.

## Acknowledgements

This work was supported by the Japan Society for the Promotion of Science [JSPS KAKENHI Grant-in-Aid for Scientific Research (C): 20K07927].

### Conflicts of interest

K.H. has received lecture honoraria for Dainippon-Sumitomo, Eisai, Janssen, Meiji Seika, MSD, and Otsuka within the past 3 years. C.U. has received lecture honoraria for Shionogi, Eisai, and MSD, and consultation fees for Ono and TONIX within the past 3 years. H.N. declares that he has no conflicts of interest.

## References

[R1] American Psychiatric Association (2013). Diagnostic and statistical manual of mental disorder. 5th ed. American Psychiatric Publishing.

[R2] Burton JK Craig LE Yong SQ Siddiqi N Teale EA Woodhouse R. (2021). Non-pharmacological interventions for preventing delirium in hospitalised non-ICU patients. Cochrane Database Syst Rev 7:CD013307. 34280303 10.1002/14651858.CD013307.pub2 PMC8407051

[R3] Charlson ME Pompei P Ales KL MacKenzie CR (1987). A new method of classifying prognostic comorbidity in longitudinal studies: development and validation. J Chronic Dis 40:373–383. 3558716 10.1016/0021-9681(87)90171-8

[R4] European Delirium Association and American Delirium Society (2014). The DSM-5 criteria, level of arousal and delirium diagnosis: inclusiveness is safer. BMC Med 12:141. 25300023 10.1186/s12916-014-0141-2 PMC4177077

[R5] Falkson G Von Hoff D Klaassen D Du Plessis H Van Der Merwe CF Van Der Merwe AM Carbone PP (1980). A phase II study of neocarzinostatin (NSC 157365) in malignant hepatoma. An Eastern cooperative oncology group pilot study. Cancer Chemother Pharmacol 4:33–36. 6244904 10.1007/BF00255455

[R6] Hatta K Kishi Y Wada K Takeuchi T Odawara T Usui C Nakamura H; DELIRIA-J Group (2014). Preventive effects of ramelteon on delirium: a randomized placebo-controlled trial. JAMA Psychiatry 71:397–403. 24554232 10.1001/jamapsychiatry.2013.3320

[R7] Hatta K Kishi Y Wada K (2015). Ramelteon for delirium in hospitalized patients. JAMA 314:1071–1072. 26348758 10.1001/jama.2015.8522

[R8] Hatta K Kishi Y Wada K Takeuchi T Ito S Kurata A; DELIRIA-J Group (2017). Preventive effects of suvorexant on delirium: a randomized placebo-controlled trial. J Clin Psychiatry 78:e970–e979. 28767209 10.4088/JCP.16m11194

[R9] Hatta K Kishi Y Wada K Takeuchi T Hashimoto N Suda K. (2019). Real-world effectiveness of ramelteon and suvorexant for delirium prevention in 948 patients with delirium risk factors. J Clin Psychiatry 81:19m12865. 10.4088/JCP.19m12865 31851436

[R10] Hughes CP Berg L Danziger WL Coben LA Martin RL (1982). A new clinical scale for the staging of dementia. Br J Psychiatry 140:566–572. 7104545 10.1192/bjp.140.6.566

[R11] Iwata N Ishigooka J Naoi I Matsumoto M Kanamori Y Nakamura H Higuchi T (2020). Long-term safety and efficacy of blonanserin transdermal patches in japanese patients with schizophrenia: a 52-week open-label, multicenter study. CNS Drugs 34:103–116. 31883082 10.1007/s40263-019-00692-6 PMC6982629

[R12] Kato M Kishi Y Okuyama T Trzepacz PT Hosaka T (2010). Japanese version of the delirium rating scale, revised-98 (DRS-R98-J): reliability and validity. Psychosomatics 51:425–431. 20833942 10.1176/appi.psy.51.5.425

[R13] Khaing K Nair BR (2021). Melatonin for delirium prevention in hospitalized patients: a systematic review and meta-analysis. J Psychiatr Res 133:181–190. 33348252 10.1016/j.jpsychires.2020.12.020

[R14] Liu Y Li XJ Liang Y Kang Y (2019). Pharmacological prevention of postoperative delirium: a systematic review and meta-analysis of randomized controlled trials. Evid Based Complement Alternat Med 2019:9607129. 31001357 10.1155/2019/9607129 PMC6437723

[R15] LONASEN Tapes®package insert. https://www.info.pmda.go.jp/go/pack/1179700S1021_1_08/. [Accessed 23 March 2022]

[R16] Maldonado JR (2018). Delirium pathophysiology: an updated hypothesis of the etiology of acute brain failure. Int J Geriatr Psychiatry 33:1428–1457. 29278283 10.1002/gps.4823

[R17] Musselman M Faden J Citrome L (2021). Asenapine: an atypical antipsychotic with atypical formulations. Ther Adv Psychopharmacol 11:20451253211035269. 34540197 10.1177/20451253211035269 PMC8442490

[R18] Ogawa A Okumura Y Fujisawa D Takei H Sasaki C Hirai K. (2019). Quality of care in hospitalized cancer patients before and after implementation of a systematic prevention program for delirium: the DELTA exploratory trial. Support Care Cancer 27:557–565. 30014193 10.1007/s00520-018-4341-8

[R19] Pendlebury ST Lovett NG Smith SC Dutta N Bendon C Lloyd-Lavery A. (2015). Observational, longitudinal study of delirium in consecutive unselected acute medical admissions: age-specific rates and associated factors, mortality and re-admission. BMJ Open 5:e007808. 10.1136/bmjopen-2015-007808 PMC4654280 26576806

[R20] Pinho C Cruz S Santos A Abelha FJ (2016). Postoperative delirium: age and low functional reserve as independent risk factors. J Clin Anesth 33:507–513. 26604015 10.1016/j.jclinane.2015.09.002

[R21] Prescribing Information of Dexmedetomidine Hydrochloride Injection. https://www.accessdata.fda.gov/drugsatfda_docs/label/2015/206628s000lbl.pdf. [Accessed 29 June 2022]

[R22] Trzepacz PT (1999). The delirium rating scale. Its use in consultation-liaison research. Psychosomatics 40:193–204. 10341531 10.1016/S0033-3182(99)71235-1

[R23] Trzepacz PT Maldonado JR Kean J Abell M Meagher DJ (2010). Delirium rating scale-revised-98 (DRS-R98) administration manual. Electronic edition. Trzepacz PT.

[R24] Wang W Li HL Wang DX Zhu X Li SL Yao GQ. (2012). Haloperidol prophylaxis decreases delirium incidence in elderly patients after noncardiac surgery: a randomized controlled trial*. Crit Care Med 40:731–739. 22067628 10.1097/CCM.0b013e3182376e4f

[R25] Xu S Cui Y Shen J Wang P (2020). Suvorexant for the prevention of delirium: a meta-analysis. Medicine (Baltimore) 99:e21043. 32791676 10.1097/MD.0000000000021043 PMC7386982

